# Magnetic Properties in Co-Deposited Iron and Metal-Free Phthalocyanine Thin Films

**DOI:** 10.3390/nano16171061

**Published:** 2026-08-26

**Authors:** Sophealena Chhom, Kevin Cano, Thomas Gredig

**Affiliations:** Department of Physics and Astronomy, California State University Long Beach, Long Beach, CA 90840, USAcanokevin123@gmail.com (K.C.)

**Keywords:** phthalocyanine, molecular thin films, magnetic dilution, low-dimensional magnetism

## Abstract

Magnetic molecular thin films provide a platform for nanoscale control of spin density, morphology and low-dimensional magnetism. We use co-deposition of closely isostructural iron phthalocyanine (FePc) and metal-free phthalocyanine (H_2_Pc) onto heated substrates to prepare diluted thin films with systematically varied Fe spin densities. Structural and surface characterization shows that H_2_Pc incorporation modifies film growth, producing a monotonic dependence of surface roughness on dilution and a grain size minimum for mixed FePc:H_2_Pc films. Vibrating sample magnetometry reveals a nonlinear suppression of the magnetic response with increasing H_2_Pc content, exceeding the reduction expected from FePc concentration alone. Below 5 K, the saturation magnetization is markedly reduced in diluted films compared with undiluted FePc, suggesting that molecular packing, Fe chain length and nanoscale morphology influence the magnetic coupling strength. These findings provide insight into FePc:H_2_Pc co-deposition as a route to chemically tunable magnetic molecular nanomaterials and highlight the importance of structurally compatible molecular dilution for magnetic sensing applications.

## 1. Introduction

Quantum sensors and spintronic devices require materials in which spin density, magnetic interactions, and coherence can be controlled [1]. Molecular spin systems are attractive for this purpose because they offer chemically tunable magnetic moments, well-defined quantum states, structural reproducibility, and compatibility with low-temperature thin-film processing. In contrast to many inorganic solid-state spin systems, molecule-based materials can be modified through chemical substitution to be assembled into thin films or heterostructures, making them promising platforms for scalable magnetic, spintronic, and quantum-device architectures [2,3,4,5]. A key challenge, however, is to control intermolecular magnetic interactions and interface-dependent scattering processes while preserving the structural and electronic properties required for device integration [6,7,8].

Transition metal phthalocyanines are ideal model systems because the molecular framework for low-Z metals remains essentially unchanged while the central metal ion tunes the spin state [9]. The transition metal phthalocyanine (MPc) with molecular formula MC_32_N_8_H_16_ is an archetypal small molecule known for its thermal and chemical stability due to its well-defined geometry and π−conjugated framework. Its planar structure supports a central metal ion (usually M^2+^) whose spin state can be systematically varied through chemical substitution: due to the almost D_4*h*_ symmetry, the molecular core spins have distinct energy levels with spins ranging from S = 0 for ZnPc, S = 1/2 in CuPc, to S = 1 in FePc, and to S = 3/2 in MnPc [10,11]. Thus, they provide a family of nearly isomorphic semiconducting molecules with spin quantum numbers ranging from nonmagnetic to highly magnetic. This combination of structural similarity and tunable spin makes MPcs particularly suitable for investigations of structural, electronic, and magnetic contributions to collective behavior.

Among planar magnetic small molecules, iron phthalocyanine (FePc) is a particularly important model system. Previous studies of FePc powders have demonstrated the quasi one-dimensional character of magnetic Fe chains, in which neighboring Fe centers interact through superexchange mediated by adjacent nitrogen atoms [2,12]. Subsequent studies of FePc thin films have shown that the magnitude and character of this coupling depend strongly on molecular spacing, crystallographic phase, and molecular orientation, demonstrating that structural organization provides a direct route for controlling the magnetic ground state [13,14,15,16,17,18,19,20]. Thus, the magnetic interactions in FePc cannot be considered solely as a property of the isolated Fe^2+^ ion; rather, they emerge from the interplay among molecular spin, intermolecular separation, substrate and molecular packing geometry.

Related behavior occurs throughout the MPc family. Manganese phthalocyanine (MnPc) is also of interest as the magnetic response is sensitive to molecular packing and orientation. Prior work on FePc and MnPc has shown orientation-dependent magnetic behavior in these systems, indicating that structural phase, molecular alignment, and substrate templating can be used to tune magnetic interactions [20,21,22,23,24,25].

In thin-film form, spins in metal phthalocyanines are typically not isolated. Intermolecular coupling, spin-spin interactions, and structural disorder can significantly affect spin dynamics and reduce coherence times [26]. Understanding and controlling intermolecular magnetic interactions remains a central challenge. One effective strategy to address this challenge is magnetic dilution, where spin-active molecules are dispersed in a nonmagnetic host matrix. Metal-free phthalocyanine, H_2_Pc with H_2_C_32_N_8_H_16_, provides a structurally compatible host that spatially separates magnetic molecules while preserving the overall molecular film environment.

Previous work on electron paramagnetic resonance (EPR) studies of spin-diluted CuPc:H_2_Pc systems have shown that molecular spin coherence can persist for relatively long times, with $T_{2}$ phase coherence times on the order of microseconds at liquid-nitrogen temperatures [26]. Thus, phthalocyanine-based thin films can support robust spin coherence under experimentally accessible conditions. Furthermore, recent progress has shown that molecular spins can be integrated into single-transistor device geometries, highlighting their potential for molecular-scale quantum electronic applications [27]. In combination with 2D materials, hybrid phthalocyanine structures are used to actively tune quantum phenomena and electronic scattering in graphene [4,28,29]. These phthalocyanine/graphene structures also form the basis for device concepts where molecular spin, charge transfer, and low-dimensional transport are engineered in scalable van der Waals heterostructures.

Other molecular platforms are also being explored for sensing and quantum-enabled technologies. For example, recent work with on-chip EPR spectroscopy showed that Fe^3+^ spin centers can be coupled sensitively to integrated quantum-device architectures [30]. Porphyrins provide another chemically tunable molecular platform, for which modification of the macrocycle, protonation state, or central metal can strongly alter adsorption, charge transfer, and sensing selectivity as shown computationally [31].

Together, these developments motivate a systematic investigation of FePc diluted in H_2_Pc as a model system for controlling magnetic-ion concentration in molecular thin films. While results on pure FePc and related transition-metal phthalocyanines have been reported, less is known about how dilution with a structurally compatible, nominally non-magnetic phthalocyanine affects film growth, nanoscale surface morphology, structural order and macroscopic magnetic response. Here, we investigate co-deposited FePc:H_2_Pc thin films with systematically varied Fe^2+^ ion concentration using atomic force microscopy, x-ray diffraction, and vibrating sample magnetometry. This approach allows us to correlate magnetic dilution with surface morphology and saturation magnetization, providing insight into how molecular packing and spin-center density influence low-dimensional magnetic interactions in FePc-based organic magnetic nanomaterials.

## 2. Materials and Methods

Commercially obtained iron phthalocyanine powder (Sigma-Aldrich, Milwaukee, WI, USA) was purified prior to thin-film deposition using the thermal-gradient sublimation procedure [32]. The commercially obtained FePc powder had a nominal purity of 90% before purification. This material was loaded into an open-ended glass source tube, which was placed inside a 25 cm long open-ended glass tube positioned within a Lindberg/Blue tube furnace. Additional glass collection tubes were placed in cooler regions of the furnace assembly to collect the sublimed material. The system was evacuated with a roughing pump to approximately $10^{-3}$ mbar during the process. The furnace temperature was initially ramped to 250 °C for 2 h, then increased to 450 °C and maintained for 42 h. During the purification process, FePc sublimed from the hot zone is redeposited in the cooler regions of the glass tubes. The purified material was collected and used for subsequent thermal evaporation.

The FePc:H_2_Pc thin films were prepared by thermal co-deposition using a recipe-controlled vacuum deposition system (Angstrom Engineering, Cambridge, ON, Canada) equipped with two Radak™ evaporation sources. Purified FePc and H_2_Pc powders were loaded into separate ceramic crucibles. Single-side-polished Si substrates were diced into 4×12 mm^2^ pieces for magnetic measurements and separate 12×12 mm^2^ pieces for structural and morphological characterization. Prior to deposition, the substrates were sequentially sonicated in acetone, methanol, and isopropanol and dried under nitrogen.

Before the deposition, the phthalocyanine sources were baked at 150 °C under closed shutters in high vacuum. Films were deposited at a base pressure of approximately $3\times 10^{-6}$ mbar. During growth, the FePc source was maintained near 350 °C and the H_2_Pc source near 300 °C and the deposition rate was monitored with two separate quartz crystal microbalances. The substrate temperature was held at 200 °C, and the sample holder was continuously rotated to improve film uniformity. At this elevated substrate temperature, large crystals with longer average chain lengths emerge that have effectively larger macroscopic magnetic signals [24]. Five nominal compositions were prepared: 100% FePc, 90:10 FePc:H_2_Pc, 75:25 FePc:H_2_Pc, 50:50 FePc:H_2_Pc, and 100% H_2_Pc. The relative deposition rates of the two sources were adjusted before opening the substrate shutter to obtain the desired mixing ratios. The deposition was stopped when the film thickness reached approximately 100 nm. The total thickness was also verified with low-angle x-ray reflectivity (XRR), see Figure S1. Successive peaks between 0.5° and 1.5° were fit to obtain a measured thickness of 96 nm to 108 nm for individual thin films.

One separate pure FePc sample was deposited at a substrate temperature of 160 °C and a base pressure of $4\times 10^{-6}$ mbar for a total deposition time of 51 min to reach about 200 nm thickness. This sample was used for time-dependent magnetic measurements yielding about twice the magnetic response due to its thickness. The measured FePc surface area was 0.358 cm^2^.

X-ray diffraction (XRD) measurements were performed with Rigaku-SmartLab X-ray diffractometer (Rigaku Corporation, Tokyo, Japan) to determine the crystalline structure and phase composition of the films. Measurements were carried out on samples with areas at least 12×12 mm^2^. The diffraction data were used to identify molecular packing, preferred crystallographic orientation, and structural changes associated with dilution by H_2_Pc.

Surface morphology was characterized using atomic force microscopy (AFM) with the Cypher S (Asylum Research - Oxford Instruments, Santa Barbara, CA, USA) in non-contact mode. Measurements were performed using high-frequency NCHR cantilevers with a resonance frequency of approximately 323 kHz and an aluminum reflective back coating. AFM images were acquired over 2 × 2 μm^2^ square areas at a fixed line scanning rate of 0.8 Hz. These measurements were used to evaluate surface roughness, grain morphology, and the evolution of film topography as a function of composition. The XRD and AFM data were analyzed using rigakuXRD and nanoAFMr packages, which provide reproducible R-based workflows for data import and image analysis [33].

Magnetic measurements were performed using the vibrating sample magnetometer (VSM) option in the PPMS (Quantum Design Inc., San Diego, CA, USA). Measurements were carried out on narrow 4×12 mm^2^ samples mounted onto a quartz holder using roughly equal lengths of Kapton tape. To limit differences associated with aging and atmospheric exposure [34], all VSM measurements were performed within one week of sample fabrication. Magnetization data were collected over an applied magnetic field range of ±1 T at 295 K, 10 K, 5 K, and 4 K. The magnetic field was applied in the film plane, i.e., parallel to the substrate surface. For some grains, the magnetic field is along the *b*-axis and for others along the projection, as the elongated grains have random directions on the surface [35]. The raw magnetic response was corrected by subtracting a room-temperature diamagnetic background and was subsequently normalized by the thin-film volume to obtain the intrinsic magnetization of the thin films.

## 3. Results

The co-deposited FePc:H_2_Pc thin-film series is characterized by combining x-ray diffraction, atomic force microscopy, and vibrating sample magnetometry to relate structural order and surface morphology to the resulting magnetic response.

### 3.1. XRD

**Figure 1:**
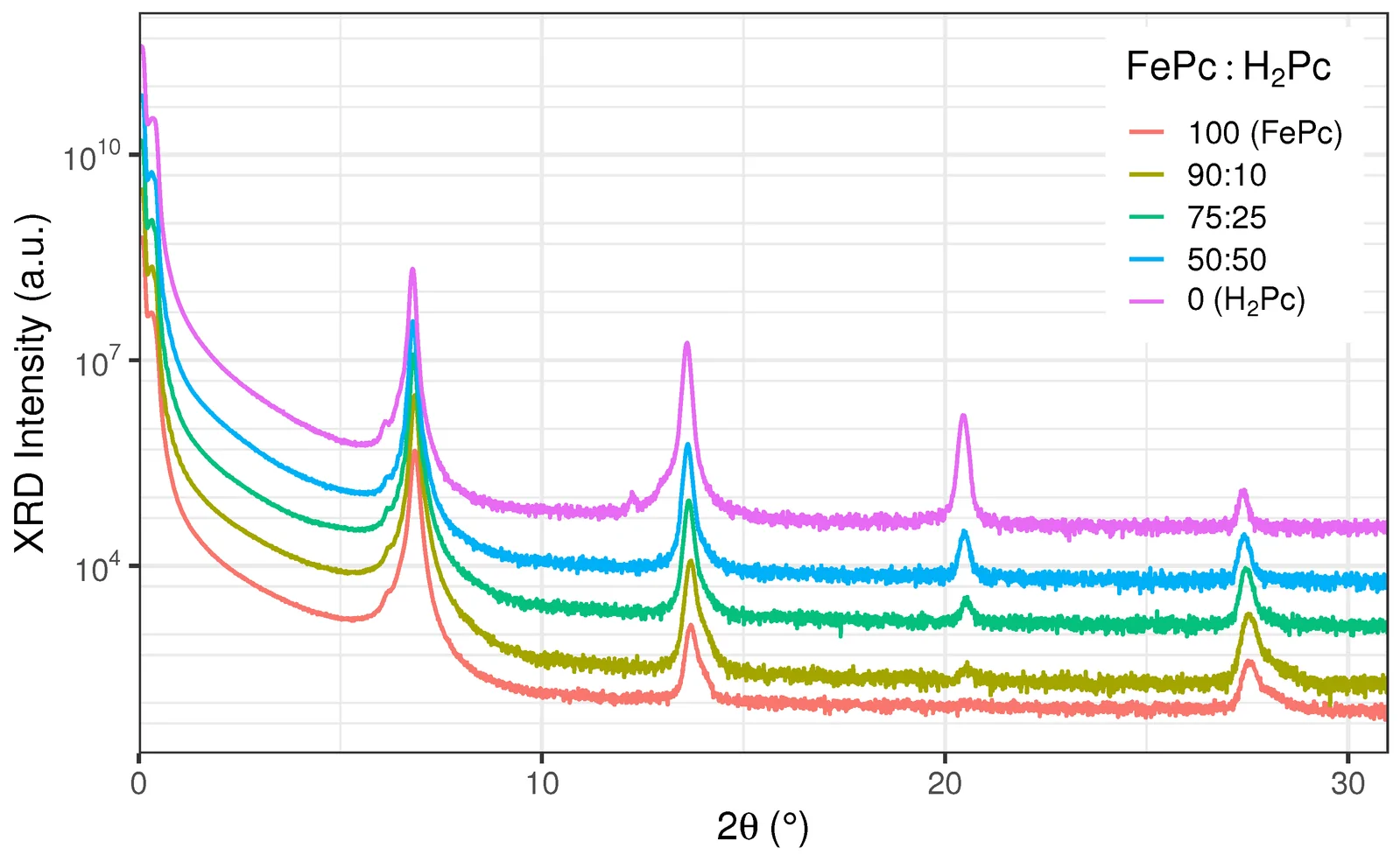
X-ray diffraction patterns of five co-deposited FePc:H_2_Pc thin films with FePc content varying from 100% to 0%. The top-most curve corresponds to pure H_2_Pc. The diffraction profiles are vertically offset for clarity. The suppressed peak near 2*θ* = 20.5° in Fe-rich films is attributed to destructive interference between scattering from the phthalocyanine macrocycle and the central Fe ion. The fitted peak positions for the H_2_Pc sample are at 2*θ* = 6.79°, 13.60°, 20.45°, and 27.39°.

**Figure 2:**
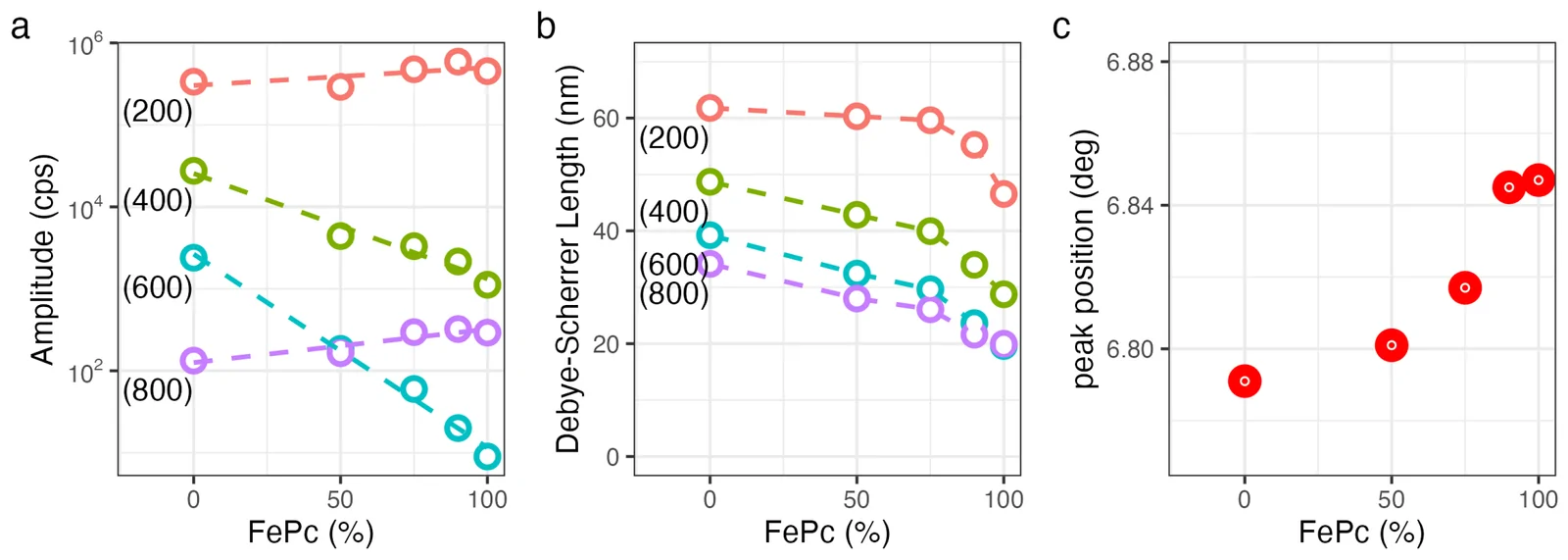
Analysis of the x-ray diffraction data for co-deposited FePc:H_2_Pc thin films. (**a**) XRD peak amplitude as a function of composition, showing peak-dependent changes in diffraction intensity. (**b**) Debye–Scherrer coherence length extracted from the XRD peak width. (**c**) Diffraction peak position as a function of FePc:H_2_Pc composition. The lines are guides to the eye.

The presence of diffraction peaks over multiple orders indicates oriented crystalline growth in all co-deposited thin films, see Figure 1. These planar molecules can self-assemble in different configurations on various surfaces [5,24,36]. The prominent x-ray diffraction peaks near 6.8° confirm the edge-on or standing configuration on silicon substrates for all samples in the series. We attribute the growth phase to the triclinic unit cell in the α-phase for FePc [37]. For the FePc-rich films, these reflections are indexed as the FePc (200), (400), (600), and (800) peaks. Since H_2_Pc adopts a similar standing molecular configuration, the corresponding diffraction peaks are observed across the dilution series with small composition-dependent shifts, see Figure 2c.

In H_2_Pc, the electron density is distributed primarily over the carbon-nitrogen macrocycle, whereas in FePc the central Fe ion introduces an additional localized electron-density maximum at the molecular center. This central scattering contribution does not enhance all reflections uniformly; for the second and third peaks, scattering from the Fe center is out of phase with part of the scattering from the surrounding carbon-rich phthalocyanine ring. The resulting destructive interference in the molecular structure factor is understood as a suppression of the FePc intensities relative to H_2_Pc. To clearly show this, we extracted the amplitudes from a Gaussian curve fit and graphed each peak for all dilutions in Figure 2a. For reflections with Miller indices (200) and (800), the amplitude remains almost constant over the series, whereas the amplitudes of the (400) and (600) peaks strongly decrease with increasing FePc content. This observation is consistent with earlier calculations and experimental measurements [38]. Liu et al. attribute the suppression of the second-order diffraction peak to destructive interference between scattering from the central atom and the surrounding phthalocyanine macrocycle. Their structure factor calculations show that the first- and second-order peak intensities evolve in opposite directions as the atomic number of the central ion increases from the metal-free H_2_Pc limit to BaPc (Z=56) [38]. For FePc (Z=26), the calculated XRD peak intensity ratio I(200)/I(400) in that analysis is expected to be around 3 to 4 times higher than for H_2_Pc. Therefore, the relative higher-order XRD intensities could be used as a rough measure of the amount of dilution.

We also measured small changes in the *d*-spacing with the dilution of Fe. The position of the FePc(200) peak for the pure FePc thin film is at 6.85°, which corresponds to a *d*-spacing of 12.9 Å; it gradually shifts towards a peak position of 6.79° in pure H_2_Pc, corresponding to a *d*-spacing of 13.0 Å, see Figure 2c. The substrate temperature also affects the *d*-spacing; higher growth temperatures reduce the *d*-spacing [39]. All films in this series were deposited on heated substrates at 200 °C, which is known to result in larger grains and more compact molecular packing in phthalocyanine films [35]. Similar to the first-order peak shift, the FePc(400) peak shifts from 13.7° towards 13.6° with FePc dilution. A strong H_2_Pc(600) peak at 20.45° is observed, but vanishes almost completely for FePc. The Debye–Scherrer coherence length is estimated from the full width at half maximum of the XRD peak. Clear trends are visible in the curves shown in Figure 2b. As expected, higher-order peaks have shorter lengths. The coherence length is smaller than the film thickness, which means crystallite domain size normal to the surface does not cover the full film thickness.

### 3.2. AFM

**Figure 3:**
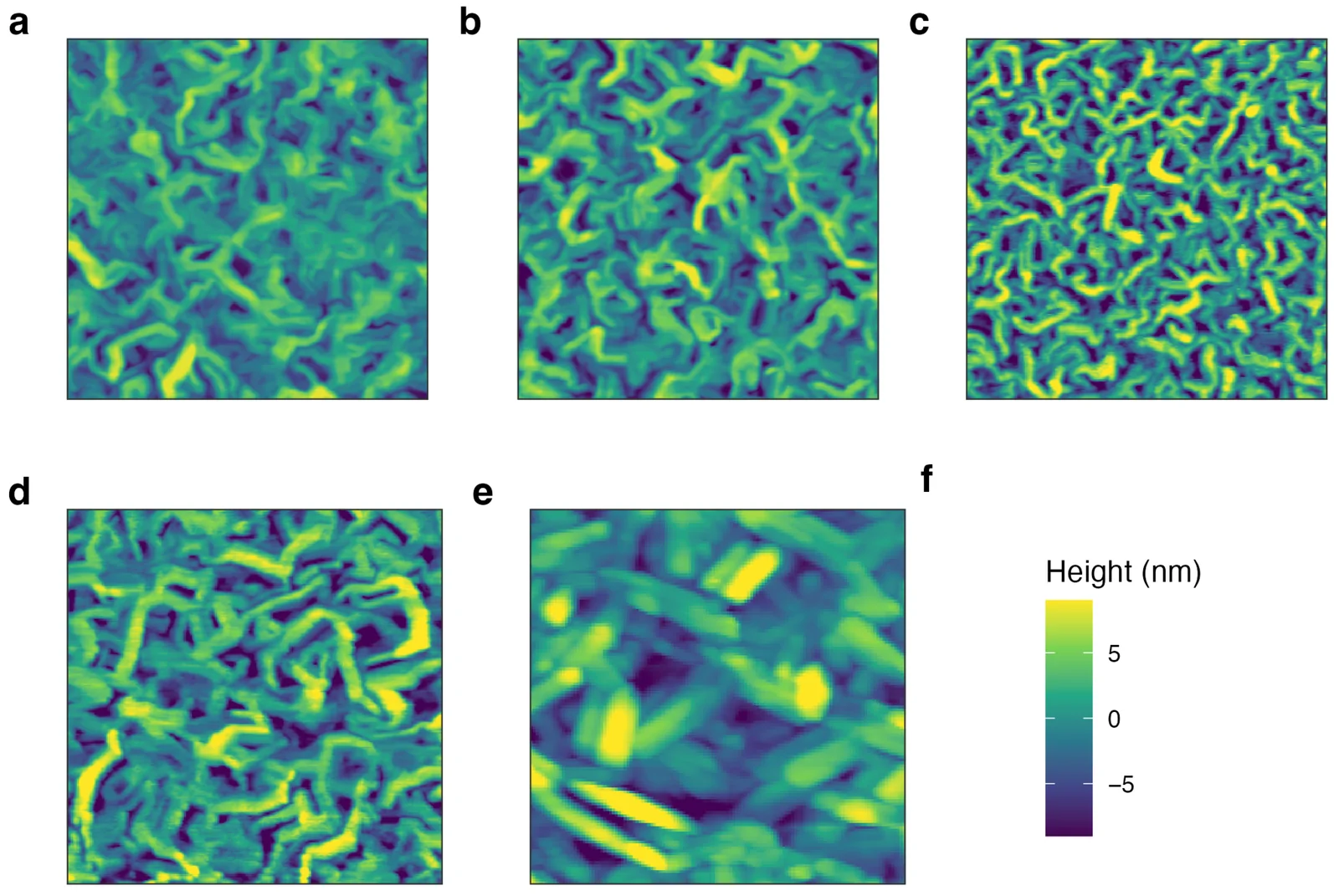
Atomic force microscopy images of co-deposited FePc:H_2_Pc thin films with FePc contents of (**a**) 100%, (**b**) 90%, (**c**) 75%, (**d**) 50%, and (**e**) 0%, corresponding to pure H_2_Pc. All AFM images are displayed with the same lateral scan size of 2000 nm. The shared height scale is shown in panel (**f**), with z=0 defined as the mean surface height for each image.

Many high-resolution atomic force microscopy (AFM) images for either pure iron phthalocyanine [35,40] or pure metal-free phthalocyanine [41] are available. The mixed phases in Figure 3, however, provide new insights into the morphology and growth of co-deposited films. Additional AFM images of size 2 × 2 μm^2^ were taken at several locations to compute average roughness values.

**Figure 4:**
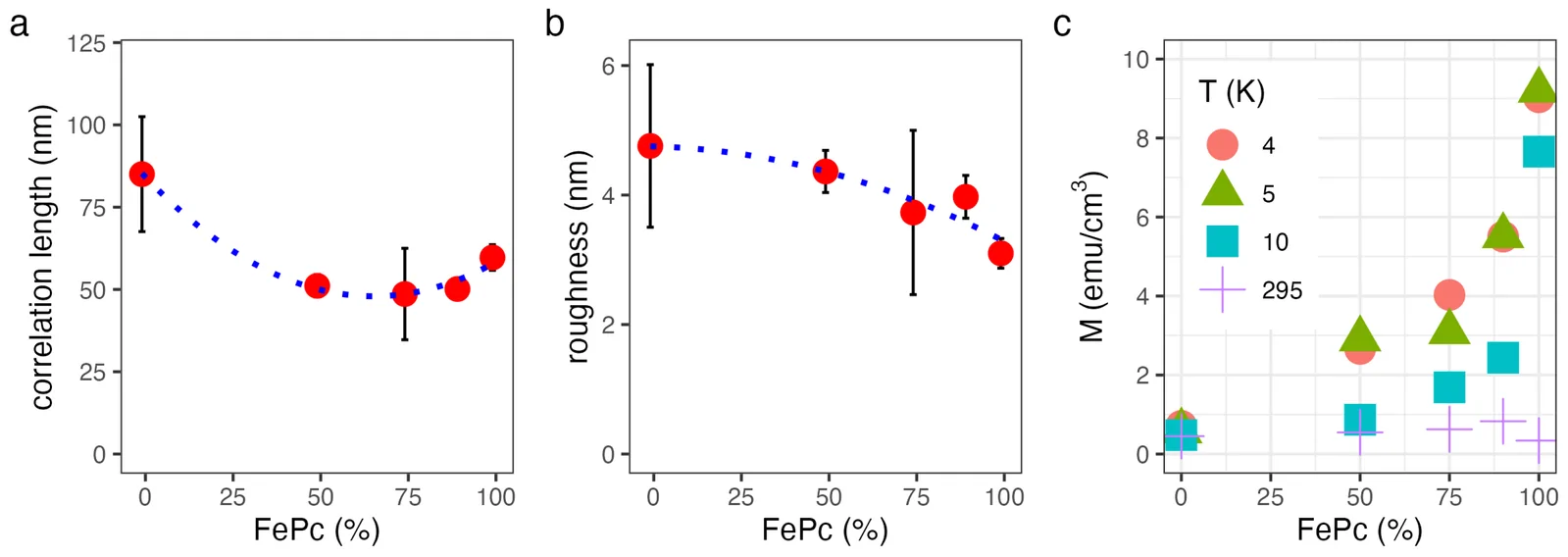
AFM and VSM analysis of co-deposited FePc:H_2_Pc thin films as a function of composition. Height–height correlation function analysis of the AFM images was used to extract (**a**) the lateral correlation length and (**b**) the surface roughness. Lines are guides to the eye. (**c**) Saturation magnetization obtained from vibrating sample magnetometry measurements is normalized by sample area and thickness and graphed for 4, 5, 10 and 295 K. Error bars are smaller than the symbol size.

The topographical data were analyzed with the height-height correlation function (HHCF) for multiple images taken at different locations on the samples [33,42]. The HHCF can be fit to a phenomenological function with three fit parameters: the long-range surface roughness, the correlation length, and the Hurst exponent, a factor that characterizes the short-range roughness. The extracted values for the correlation length and long-range surface roughness are plotted in Figure 4a,b. It is intriguing to observe the nonlinear effects of surface correlation length with the co-deposited samples. The correlation length dips from 60 nm for FePc to 49 nm for 75:25 FePc:H_2_Pc and then increases back to 85 nm for H_2_Pc. Meanwhile, the root mean square (rms) roughness increases gradually from 3.1 nm to 4.8 nm. The Hurst exponent remains constant at around 0.85 for all samples in the series.

The rms roughness and lateral correlation length in Figure 4 probe different aspects of film morphology and vary differently with dilution. The non-monotonic correlation length suggests a crossover in growth behavior: at high FePc concentrations, incorporation of H_2_Pc may disrupt FePc packing and limit lateral domain growth, consistent with enhanced nucleation or grain-boundary pinning. At low FePc concentrations, H_2_Pc-rich domains can coarsen, increasing the lateral correlation length toward that of pure H_2_Pc. Because H_2_Pc forms a rougher, more needle-like morphology than FePc, this lateral coarsening can occur concurrently with increased vertical roughness. Vertical compositional segregation may also contribute, but the present measurements do not resolve the depth- dependent composition.

### 3.3. VSM

The reduced dimensionality of FePc has important consequences for the magnetic properties. The low-dimensional magnetism differs from a conventional correlated three-dimensional magnet characterized by a Curie temperature [2]. Moreover, the volume-normalized saturation moment for an FePc thin film is approximately 80 times smaller than that of a metallic Fe thin film. The precise value depends on a few factors, including growth conditions, unit cell and molecular packing, and exposure to air, oxygen and moisture [19]. Subsequently, magnetization curves yield low magnetization values.

**Figure 5:**
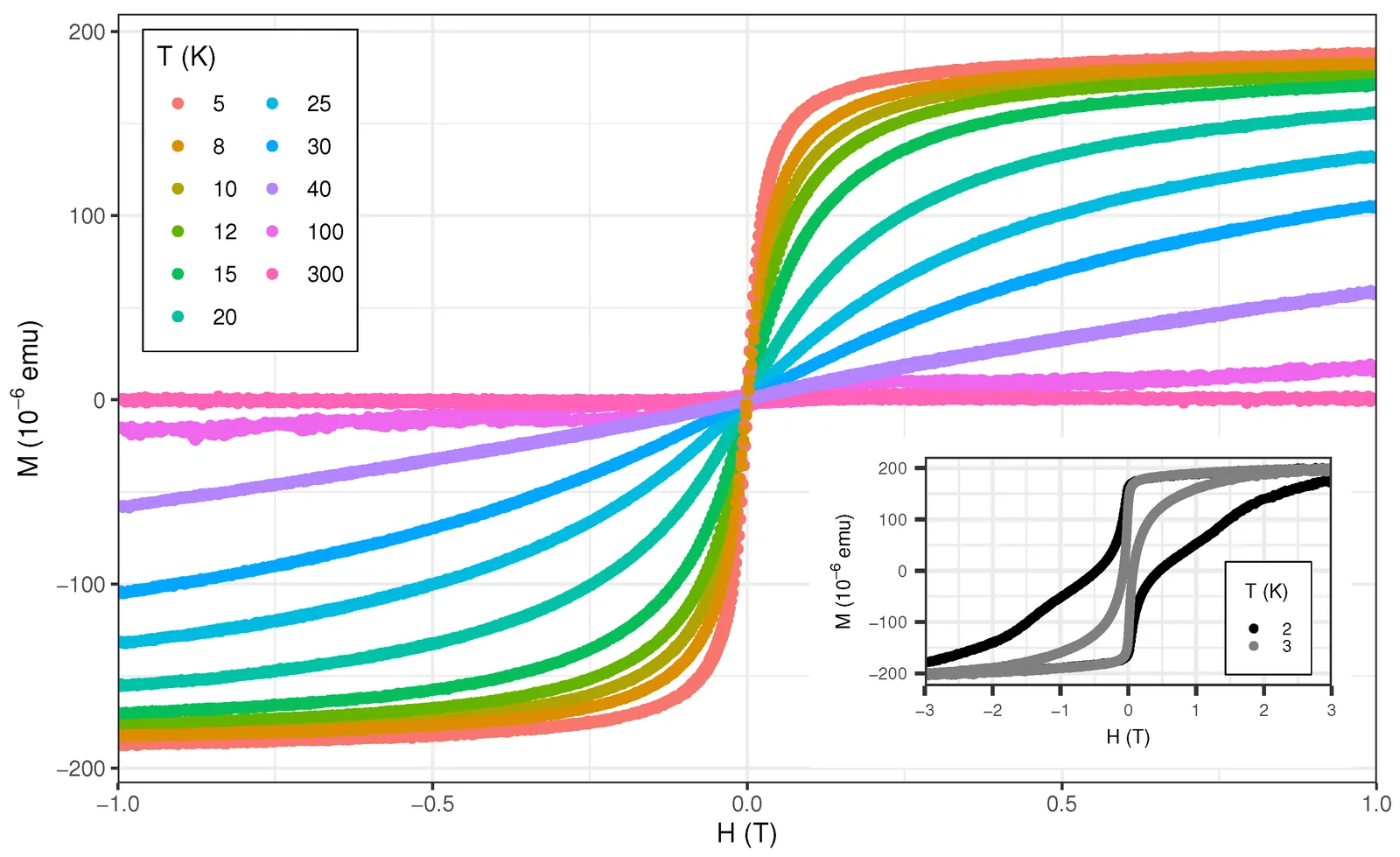
Magnetization versus applied magnetic field curves for a representative 200 nm thick FePc film deposited at 160 °C. Below 5 K, the magnetization curves exhibit clear hysteresis with large coercive fields. At the lowest temperatures, applied fields of up to 5 T are required to fully saturate the film. The coercivity can reach almost 1 T in magnitude for some FePc samples.

The temperature dependence of the magnetization curves from room temperature to 2 K is shown for a 200 nm thick FePc thin film in Figure 5. Each magnetization curve was measured with the same sweep speed of 20 Oe/s. Slower sweep speeds result in less coercivity. The diamagnetic signal of $-5.4\times 10^{-9}$ emu/Oe from the substrate has been removed for all curves. Below 20 K, saturation for applied fields of 1 T is observed, similar to reports for α-phase FePc powder (near 25 K) [12]. Below 5 K, the magnetization becomes hysteretic with some very large coercive values for samples with large grain sizes. Coercive fields of up to 1 T have been measured in FePc thin films. The shape resembles an hourglass with the center pinched. The coercivity values depend on both the deposition temperature and the sweep speed of the applied magnetic field [22].

**Figure 6:**
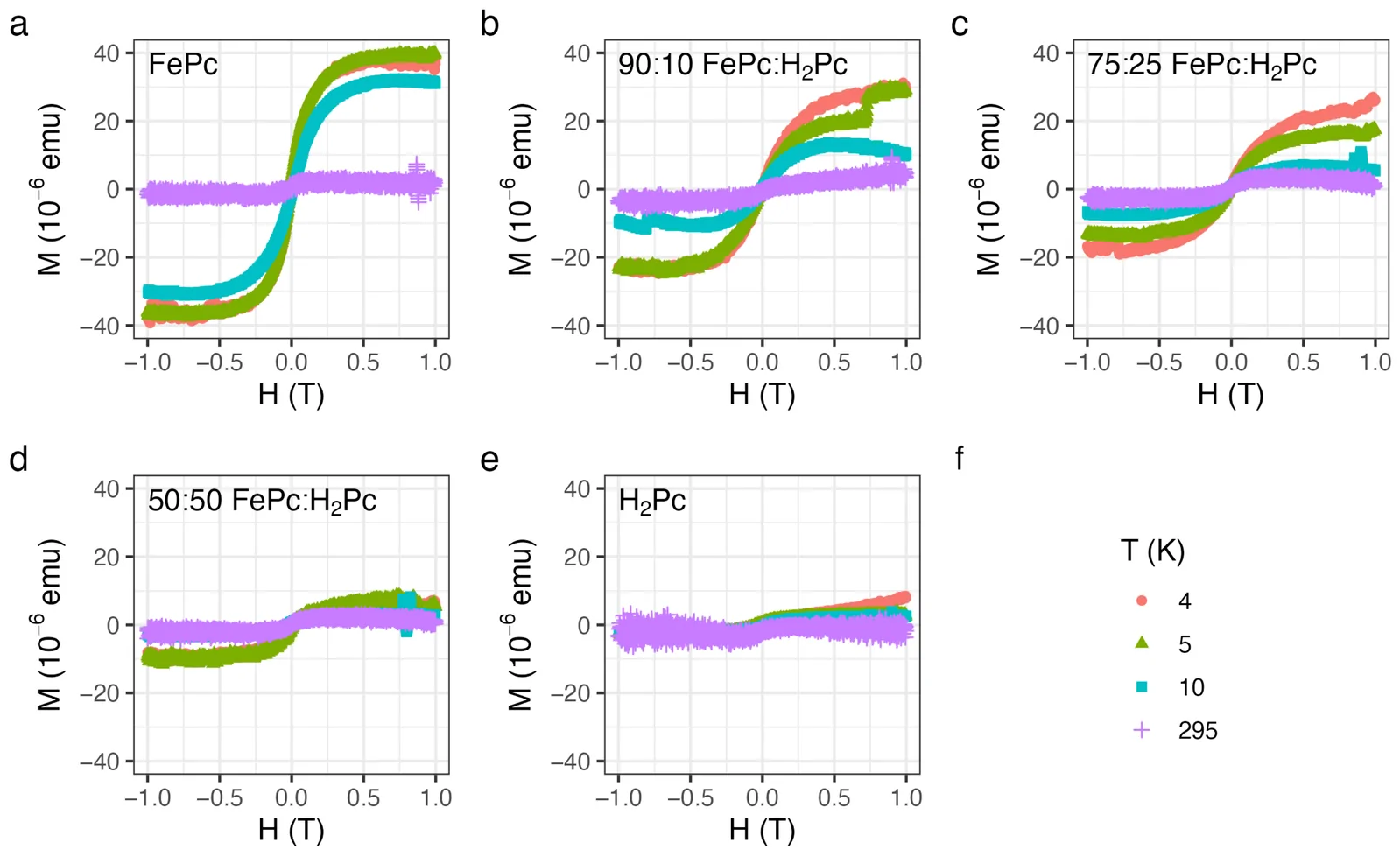
Magnetization versus applied magnetic field curves for five co-deposited FePc:H_2_Pc thin films with FePc contents of (**a**) 100%, (**b**) 90%, (**c**) 75%, (**d**) 50%, and (**e**) 0%, corresponding to pure H_2_Pc. Panel (**f**) provides the legend for the measurement temperatures: 4 K, 5 K, 10 K, and room temperature. The data show the nonlinear evolution of the magnetic response with composition. For each sample, the diamagnetic contribution from the corresponding Si substrate was subtracted.

The magnetic response from the co-deposited FePc:H_2_Pc samples are plotted in Figure 6. Due to the small magnetic response of metal-organic thin films for that thickness, a small contribution from impurities is observed even at room temperature [43]. The low-temperature response is clearly distinguished for samples with higher Fe ion concentrations.

For the H_2_Pc film in Figure 6e, the magnetic signal at room and low temperature is within the uncertainty; thus, we observe no extra magnetic response from this sample. Conversely, for the pure FePc film in Figure 6a, the low-temperature response is strong at 10 K and peaks at the lowest temperatures. Strikingly, the macroscopic magnetic response does not scale linearly with the amount of FePc in the thin film, see Figure 4c; i.e., for the sample with half the FePc material, the signal is about one-quarter of the full magnetic response, ∼10 μemu compared with 40 μemu. This nonlinear magnetic response could be related to shortening or interruption of the quasi-one-dimensional FePc spin chains. Vargas et al. showed that finite FePc chains can exhibit a non-collinear, helical spin structure, illustrating that the magnetic response depends sensitively on chain length and boundary conditions [44]. Theoretical efforts to describe the spin structure and correlations in low-dimensional FePc systems are also ongoing [45]. Within this context, the VSM magnetization curves in Figure 6 show that the diluted samples are not merely scaled-down versions of pure FePc. Instead, the nonlinear decrease in magnetization with FePc concentration is consistent with a reduction in the number of 1D-connected Fe spin centers. To illustrate how dilution could modify this connectivity, we consider a simple one-dimensional site-dilution model. It can be used to estimate the effect of H_2_Pc incorporation on the FePc correlation length. Note that the FePc correlation length in diluted samples must be smaller than the measured correlation length from AFM in Figure 4a. Let *p* denote the probability that a molecular site along the 1D chain is occupied by Fe^2+^. The probability of maintaining an uninterrupted one-dimensional sequence of FePc molecules over *n* sites then scales as $p^{n}$. The uninterrupted chain has a connection length of na, where *a* is the ion-ion distance along the chain. Writing this decay in exponential form (exp(nlnp)) gives a dilution-limited characteristic length $\xi_{Fe}\left(p\right)=-a/\ln p$. For a pure FePc film (p→1), $\xi_{Fe}$ is limited by the crystallite or sample dimensions, whereas $\xi_{Fe}\to 0$ for high dilution (p→0). This model gives $\xi_{Fe}\approx 9.5a$, 3.5a, and 1.4a for FePc concentrations of 90%, 75%, and 50%, respectively. The model could qualitatively describe the macroscopic VSM measurements; however, we did not directly determine the microscopic Fe chain length or changes in the exchange coupling of the diluted FePc thin films. A more detailed test would require additional compositions and a local or element-specific magnetic probe.

Using the sample area and individual film thickness, we obtain a normalized saturation value of approximately 10 emu/cm^3^, see Figure 4c. We note that this result is slightly lower than previously reported values in this material [19,24]. The variation can be explained by known factors that affect the absolute value, including molecular alignment, substrate-templated growth and oxygen absorption through air exposure or moisture. Keeping these factors consistent in the co-deposited sample series, the relative values are still relevant. Thus, co-deposited FePc:H_2_Pc thin films provide a new avenue towards tuning the spin exchange and controlling the magnetic behavior of easy-to-process small molecules.

## 4. Conclusions

We have investigated co-deposited FePc:H_2_Pc thin films as magnetically diluted molecular thin-film systems. The films retain the standing phthalocyanine packing geometry on Si substrates, with only small changes in *d*-spacing across the dilution series. Higher-order XRD peak intensities exhibit a systematic dependence on FePc:H_2_Pc composition. In particular, FePc exhibits suppressed second- and third-order diffraction peaks, consistent with destructive interference between scattering from the central Fe ion and the surrounding phthalocyanine macrocycle. AFM measurements show that dilution modifies the lateral surface morphology and affects both surface roughness and grain size. The shortest correlation length is observed for films containing a 75:25 FePc:H_2_Pc ratio. Magnetic measurements reveal that the low-temperature response is dominated by FePc and is strongly reduced by H_2_Pc dilution. The magnetization does not scale linearly with FePc content, indicating that dilution changes the magnetic exchange rather than simply reducing the number of Fe spin centers. This behavior is consistent with interruption or shortening of quasi one-dimensional Fe–N–Fe superexchange pathways. Overall, these results show that FePc:H_2_Pc co-deposition produces distinct molecular magnetic systems with modified spin exchange, providing a route toward chemically controlled organic-based magnetic thin films for spintronic and magnetic-sensing applications. Ultimately, measurements of spin-relaxation and coherence times, together with transport studies in device geometries, could establish whether molecular dilution can be used to engineer the spin density, exchange coupling, and coherence as key parameters for spintronic and quantum-sensing technologies.

## Data Availability

The original contributions presented in this study are included in the article/supplementary material. Further inquiries can be directed to the corresponding author.

## Supplementary Materials

The following supporting information can be downloaded at: https://www.mdpi.com/article/10.3390/nano16171061/s1, Figure S1: X-ray reflectivity (XRR) profiles for five samples spanning the FePc:H_2_Pc dilution series.
