# Supplementary material for: Magnetic Properties in Co-Deposited Iron and Metal-Free Phthalocyanine Thin Films
Source: Nanomaterials (Basel). 2026 Aug 26;16(17):1061. doi: 10.3390/nano16171061 (PMC13567462; doi:10.3390/nano16171061)
Supplement: Supplementary file 1 [file nanomaterials-16-01061-s001.zip › nanomaterials-4446338-supplementary.pdf]

Supplemental Materials: Magnetic Properties in Co-Deposited Iron and Metal-Free Phthalocyanine Thin Films

Sophealena Chhom, Kevin Cano, Thomas Gredig

Department of Physics and Astronomy, California State University Long Beach, Long Beach, CA - USA.

2026-08-25

X-ray Reflectivity Data

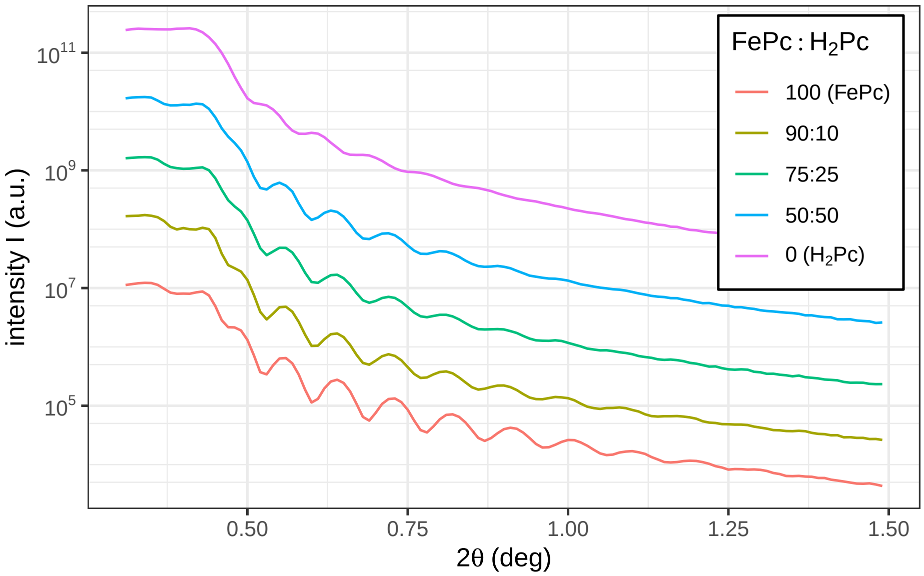

Figure S1: X-ray reflectivity (XRR) profiles for five samples spanning the FePc:H_2Pc dilution series. The curves are vertically offset for clarity.

From the oscillations in Figure S1, the following thicknesses are obtained: 96.2 nm (pure FePc), 98.7 nm (90:10 FePc:H_2Pc), 105 nm (75:25 FePc:H_2Pc), 101 nm (50:50 FePc:H_2Pc) and 108 nm (pure H_2Pc).
